# Supplementary material for: Transversus Abdominis Plane Block as a Strategy for Effective Pain Management in Patients with Pain during Laparoscopic Cholecystectomy: A Systematic Review
Source: J Clin Med. 2022 Nov 22;11(23):6896. doi: 10.3390/jcm11236896 (PMC9735918; doi:10.3390/jcm11236896)
Supplement: Supplementary file 1 [file jcm-11-06896-s001.zip › jcm-1942426-supplementary.pdf]

Table S1. Within Group Effects of the Interventions.

No	Authors	Year	Sample Size (*n*)	Time	Baseline M(SD)	Intraoperative M(SD)	Post-Operative M(SD)	Mean Analgesia Demand per Group	Level of Significance
1	El-Dawlatly [59]	2009							
	US-TAP Block		21	24 h		0.9(0.7)	10.5(7.7)	9.6	<0.05 *
						8.6(3.5)			<0.01 *
	Control (GA only)		21	24 h		2.3(1.0)	22.8(4.3)	20.5	<0.05 *
						23.0(4.8)			<0.01 *
2	Ra [7]	2010							
	US-TAP Block (0.25%)		18	20 min			3.3(1.84)	−1.2	<0.001 *
				30 min			2.9(1.68)	−0.8	
				60 min			2.8(1.70)	−0.7	
				6 h			3.1(1.55)	−1.0	
				12 h			2.1(1.41)		
				24 h			1.6(1.46)		
	US-TAP Block (0.5%		18	20 min			3.2(1.89)	−1.4	<0.001 *
				30 min			2.8(1.38)	−1.0	
				60 min			2.6(1.46)	−0.8	
				6 h			1.9(1.43)	−0.1	
				12 h			1.8(1.16)		
				24 h			1.3(0.97)		
	Control (GA only)		18	20 min			8.6(1.76)	−4.2	<0.001 *
				30 min			8.2(1.72)	−3.8	<0.001 *
				60 min			6.9(1.57)	−2.5	<0.001 *
				6 h			6.1(1.98)	−1.7	<0.001 *
				12 h			5.1(2.01)	−0.7	<0.001 *
				24 h			4.4(1.68)		<0.001 *
3	Ortiz [20]	2012							
	US-TAP Block, Bilateral		39	24 h	1.5(1.8)		16.1(9.9)	14.6	0.75
				24 h		5.0(3.5)	16.1(9.9)	11.1	
						237(82)			0.67
				24 h			1.8(1.6)		
	Control (Port Sites Infiltration)		35	24 h	0.9(2.0)		15.4(9.2)	14.5	0.75
						5.0(3.5)	15.4(9.2)	10.4	
				24 h		5.8(2.8)	15.4(9.2)	9.6	
						245(75)			0.67
				24 h			1.8(1.8)		
4	Petersen [21]	2012							
	US-TAP Block (Ropivacaine)			24 h		Ketomidone consumption $p = 0.41$, 95% CI: 0.13, 0.65			
				0–2 h			5mg		
	US-TAP Block (normal saline)					Ketomidone consumption $p = 0.68$, 95% CI: 0.45, 0.83			
				0–2 h			7.5mg		
5	Tolchard [60]	2012		1 & 4 h					
	US-STA Block		21				0.58(0.47)		0.5
							2.2(0.94)		0.56
							3.0(0.94)		0.85
							0.65(0.19)		0.18
							16(7.0)		0.61
							1.1(0.47)		
	Control (Port Site Infiltration)		22				0.65(0.46)		0.5
							2.4(0.92)		0.56
							3.1(1.4)		0.85

						0.7(3.2)		0.18
						21(9.2)		0.61
						1.2(0.46)		
6	Bhatia [6]	2014						
	US-TAP Block, Posterior		21					
				0 h		1.2(1.1)	−0.4	
				2 h		1.6(1.5)	−0.8	
				4 h		1.8(1.2)	−1.0	
				6 h		1.6(1.3)	−0.8	0.04 *
				8 h		2.2(1.8)	−1.4	0.025 *
				12 h		1.8(2.0)	−1.0	0.04 *
				24 h		0.8(0.8)		0.003 *
	US-TAP Block, Subcostal		21					
				0 h		0.85(0.87)	−0.7	
				2 h		0.8(0.89)	−0.65	
				4 h		0.8(1.2)	−0.65	
				6 h		0.9(1.1)	−0.75	0.04 *
				8 h		1.0(1.5)	−0.85	0.025 *
				12 h		0.8(1.5)	−0.65	0.04 *
				24 h		0.15(0.36)		0.003 *
	Control (GA only)		22					
				0 h		2.35(1.3)	−1.0	0.005 *
				2 h		3.0(1.3)	−1.7	0.004 *
				4 h		2.3(0.9)	−1.0	
				6 h		2.0(1.1)	−0.7	
				8 h		2.6(1.7)	−1.3	
				12 h		1.9(1.5)	−0.6	
				24 h		1.3(1.0)		
7	Shin [61]	2014						
	US-TAP-Block		15		8.0(13.7)	8.0(13.7)		0.415
					72.4(4.6)	10.0(15.8)	−62.4	0.005 *
						7.3(7.0)		0.852
					VNRS Scores at rest			
				10 min		4.3(0.5)	−2.2	0.001
				30 min		3.5(0.4)	−1.4	0.001
				1 h		3.5(0.4)	−1.4	0.001
				3 h		3.3(0.3)	−1.2	0.002
				6 h		3.6(0.3)	−1.5	0.009
				24 h		2.1(0.2)		0.014
					VNRS scores while coughing			
				10 min		4.7(0.6)	−1.8	0.001
				30 min		4.5(0.5)	−1.6	0.001
				1 h		4.5(0.5)	−1.6	0.001
				3 h		4.4(0.4)	−1.5	0.001
				6 h		4.7(0.4)	−1.8	0.003
				24 h		2.9(0.2)		0.020
	US-OSTAP		15			8.0(13.7)		0.415
					78.1(4.6)	6.7(11.4)	−71.4	0.005 *
						8.0(7.7)		0.852
					VNRS Scores at rest			
				10 min		2.3(0.5)	−1.0	0.001 *
				30 min		2.8(0.6)	−1.5	0.001 *
				1 h		2.1(0.4)	−0.8	0.001 *
				3 h		2.0(0.3)	−0.7	0.002 *
				6 h		2.3(0.3)	−1.0	0.009 *
				24 h		1.3(0.2)		0.014 *
					VNRS scores while coughing			
				10 min		2.9(0.6)	−0.8	0.001 *
				30 min		3.3(0.6)	−1.2	0.001 *
				1 h		2.7(0.4)	−0.6	0.001 *
				3 h		2.7(0.3)	−0.6	0.001 *
				6 h		3.2(0.3)	−1.1	0.003 *
				24 h		2.1(0.2)		0.020 *
	Control (GA only)		15			14.0(5.5)		0.415
					82.9(4.4)	41.7(37.4)	−41.2	0.005 *
						6.7(8.2)		0.852
					VNRS Scores at rest			
				10 min		6.6(0.5)	−4.5	0.001 *
				30 min		6.3(0.5)	−4.2	0.001 *

				1 h			5.1(0.3)	−3.0	0.001 *
				3 h			3.7(0.5)	−1.6	0.002 *
				6 h			3.3(0.3)	−1.2	0.009 *
				24 h			2.1(0.4)		0.014 *
					VNRS scores while coughing				
				10 min			7.5(0.5)	−4.5	0.001 *
				30 min			7.4(0.5)	−4.4	0.001 *
				1 h			6.4(0.4)	−3.4	0.001 *
				3 h			4.7(0.5)	−1.7	0.001 *
				6 h			4.3(0.4)	−1.3	0.003 *
				24 h			3.0(0.4)		0.020 *
8	Basaran [62]	2015							
	US-OSTAP-Block Group		38						
					19.4(47.6)				0.437
					0.26(1.6)				0.48
				0–2h			31.6(31.7)		0.001 *
				2–24 h			126.3(54.2)		0.001 *
				0–24 h			2.3(3.7)		0.719
	Control (GA only)		38						
					19.4(47.6)				0.437
					0.26(1.6)				0.48
				0–2 h			80.3(50.1)		0.001 *
				2–24 h			267.1(108.6)		0.001 *
				0–24 h			3.3(5.5)		0.719
9	Elamin [63]	2015							
	LAP-TAP Block with Bupivacaine plus Periportal saline injection.		40	In the LAP-TAP group (Bupivacaine + Periportal saline injection), numerical pain assessment scores were significantly decreased after 1, 3 and 6 h of rest (*p* = 0.025, *p* = 0.03, and *p* = 0.007, respectively).					
	LAP-TAP Block with saline plus Periportal Bupivacaine injection.		40	In the LAP-TAP group (Bupivacaine + Periportal saline injection), numerical pain assessment scores were significantly decreased after 1, 3 and 6 h of rest (*p* = 0.025, *p* = 0.03, and *p* = 0.007, respectively).					
10	Saliminia [64]	2015			Time of first Fentanyl request				
	US-TAP Block with Bupivacaine + Normal saline		18	24 h usage	144.44 (83.1)		566.7 (367.8)	422.3	0.019 *
	US-TAP Block (Bupivacaine + Sufentanil)		18	24 h usage	206.38 (112.7)		555.5 (356.8)	349.1	0.002 *
	US-TAP Block (normal saline)		18	24 h usage	79.44 (42.2)		877.8(338.8)	978.4	0.012 *
11	Al-refaey [65]	2016							
	US-TAP Block with Bupivacaine		30	Post-operative day 1			2.1(0.5)		<0.001 *
						0.5± 0.1mg			<0.011 *
	US-TAP Block with Bupivacaine + magnesium		30				2.2(0.5)	<0.001 *	
						2.1± 0.1mg			<0.011 *
	Control (GA only)		30				2.8(0.6)		<0.001 *
						0.9± 0.1mg			<0.011 *
12	Bava [66]	2016			A total of 8 patients in the US-TAP group and 16 patients in the local infiltration group required additional Fentanyl intraoperatively (*p* = 0.028). In the immediate postoperative period, local infiltration levels were significantly higher at rest and during cough (*p* = 0.034 and *p* = 0.007, respectively).				
							Median (minimum-maximum)		
	US-TAP Block		21	0 h			5.0(0–8)		0.034 *
				1 h			3.0 (2–7)		0.209
				2 h			2.0(0–5)		0.579
				4 h			2.0 (0–3)		0.864
				6 h			2.0(0–5)		0.528
				12 h			1.0(0–5)		0.176
				24 h			2.0 (0–5)		0.01 *
							34.57 x 14.64 mg		0.688
	Control (Port Sites infiltration)		21	0 h			7.0 (2–8)		0.034 *

				1 h	2.0 (0–8)		0.209
				2 h	2.0 (0–6)		0.579
				4 h	2.0 (0–7)		0.864
				6 h	1.0 (0–5)		0.528
				12 h	2.0 (0–6)		0.176
				24 h	0.5 (0–5)		0.01 *
					32.76 x 14.34 mg		0.688
13	Huang [67]	2016					
	US-TAP Block		20	0	2.8(1.3) *	−1.1	
				30 min	2.6(1.4) *	−0.9	
				60 min	2.5(1.3) *	−0.8	
				6 h	2.9(1.5) *	−1.2	
				12 h	2.3(1.5) *	−0.6	
				24 h	1.7(1.3) *		
	US-TAP Block + Dexamethasone		20	0	2.6(1.2) *	−1.1	
				30 min	2.7(1.4) *	−1.2	
				60 min	2.5(1.3) *	−1.0	
				6 h	2.6(1.4) *	−1.1	
				12 h	1.9(1.2) *	−0.4	
				24 h	1.5(1.1) *		
	Control (GA)		20	0	8.5(1.6)	−4.3	
				30 min	6.5(1.1)	−2.3	
				60 min	6.5(1.5)	−2.3	
				6 h	6.3(1.4)	−2.1	
				12 h	5.6(1.6)	−1.4	
				24 h	4.2(1.4)		
14	Oksar [68]	2016					
	Intercostal-iliac US-TAP Block		22		11(55%) do not require additional analgesics; 9(45%) require additional analgesics.		
	US-OSTAP Block		22		17(85%) do not require additional analgesics; 3(15%) require additional analgesics.		
	Control (GA + PCA only)		22		7(35%) do not require additional analgesics; 13(65%) require additional analgesics.		
15	Sinha [69]	2016			Median IQR		
	US-TAP Block with 0.25% Bupivacaine		30	10 min	0.00(0.00–0.00)		0.044 *
				30 min	1.00(0.00–1.00)		0.003 *
				1 h	1.50(0.75–2.25)		0.020 *
				4 h	2.00(1.00–5.00)		0.441
				8 h	2.00(1.00–2.00)		0.164
				12 h	1.50(1.00–2.00)		0.803
				24 h	1.00(1.00–2.00)		1.000
	US-TAP Block with 0.375% Ropivacaine		30	10 min	0.00(0.00–0.00)		0.044 *
				30 min	0.00(0.00–0.00)		0.003 *
				1 h	0.00(0.00–2.00)		0.020 *
				4 h	2.00(1.00–4.00)		0.441
				8 h	2.00(1.00–4.25)		0.164
				12 h	0.00(0.00–2.00)		0.803
				24 h	1.00(1.00–2.00)		1.000
16	Breazu [70]	2017			Pain score at rest: Results presented as median (25th to 75th percentile)		
	US-OSTAP (Bupivacaine)			0 h	3(2–4)		
				2 h	3(3–4)		
				4 h	4(3–4)		
				6 h	2(2–3)		
				12 h	2(1–2)		
				24 h	1(1–2)		
					Pain score in movement: Results presented as median (25th to 75th percentile)		
				0 h	1(1–2)		
				2 h	1(1–3)		
				4 h	1(1–2)		
				6 h	1(0–1)		
				12 h	0(0–1)		
				24 h	0(0–1)		

					24 h	160(140–180)		
					24 h		34.8(26.94)	
			US-OSTAP (Pethidine)			Pain score at rest: Results presented as median (25th to 75th percentile)		
					0 h		1(1–2)	
					2 h		1(1–2)	
					4 h		1(1.2)	
					6 h		1(0–1)	
					12 h		0(0–1)	
					24 h		0(0–0)	
						Pain score in movement: Results presented as median (25th to 75th percentile)		
					0 h		0(0–1)	
					2 h		1(1–2)	
					4 h		1(1–2)	
					6 h		1(0–1)	
					12 h		0(0–1)	
					24 h		0(0–0)	
					24 h	150(140–180)		<0.001
					24 h		20.4(20.71)	
			US-OSTAP-Block (Normal saline)			Pain score at rest: Results presented as median (25th to 75th percentile)		
					0 h		0(0–1)	
					2 h		1(1–2)	
					4 h		1(1–2)	
					6 h		1(0–1)	
					12 h		0(0–1)	
					24 h		0(0–0)	
						Pain score in movement: Results presented as median (25th to 75th percentile)		
					0 h		3(2–4)	
					2 h		3(3–4)	
					4 h		4(3–5)	
					6 h		2(2–3)	
					12 h		2(1–2)	
					24 h		1(1–2)	
					24 h	400(320–340)		
					24 h		78(17.32)	
17	Choi [71]	2017				Number of patients requiring additional analgesics		
			US-TAP block (Inserted indwelling catheter)	34				
							4(11.8%)	0.114
							17(50.0%)	0.332
			US-TAP block + PCA	34				
							2(5.9%)	0.114
							11(32.4%)	0.332
			Control (PCA)	35				
							0(0.0%)	0.114
							15(42.9%)	0.332
18	Sahin [72]	2017				Results presented as median (range)		
			US-TAP Block (Bupivacaine + 10mls normal saline).	30				
					20 min	2.0(0–8)		0.014 *
					12 h	2.5(0–7)		0.003 *
					24 h	3.0(1–7)		<0.001 *
			US-TAP Block (20mls normal saline)	30				
					20 min	2.0(0–4)		0.014 *
					12 h	1.0(0–4)		0.003 *
					24 h	1.0(0–4)		<0.001 *
19	Baral [73]	2018						
			US-TAP, subcostal	30				
					24 h		125 ± 25.42	<0.001 *
							3.20 ± 0.84	<0.001)
			Control (Port Site Infiltration)	30				
					24 h		175 ± 25.42	<0.001 *
							1.70 ± 0.65	<0.001 *

20	Bhalekar [74]	2018						
	US-TAP block, Subcostal							
				24 h		Yes = 17(68.0)<br>No = 8(32.0)		0.002 *
						Yes = 2(8.0)<br>No = 23(92.0)		0.001 *
	US-TAP block (0.9% Normal saline)							
				24 h		Yes = 25(100.00)<br>No = 0(0.00)		0.002 *
						Yes = 24(96.0)<br>No = 1(4.0)		0.001 *
21	Sarvesh [75]	2018						
	US-TAP Block 1		30	24 h		Postoperatively, it took 485.6 min for the first analgesic to be requested in US-TAP Block 2, compared with 289.8 min in US-TAP Block 1. Patients in US-TAP block 2 consumed less cumulative morphine in the first 24 h than patients in US-TAP block 1.		0.0001 *
						Patients in US-TAP block 2 consumed less cumulative morphine in the first 24 h than patients in US-TAP block 1.		<0.00001 *
	US-TAP Block 2		30	24 h		Postoperatively, it took 485.6 min for the first analgesic to be requested in US-TAP Block 2, compared with 289.8 min in US-TAP Block 1.		0.0001 *
						Patients in US-TAP block 2 consumed less cumulative morphine in the first 24 h than patients in US-TAP block 1.		<0.00001 *
22	Suseela [76]	2018						
	US-TAP Block, Subcostal		40	1 h		0.00(0.00)	1.13	
				2 h		0.03(0.16)	1.1	0.004 *
				3 h		0.43(0.55)	0.7	<0.001 *
				6 h		1.35(0.80)	−0.22	<0.001 *
				12 h		0.93(0.86)	0.2	<0.001 *
				24 h		1.13(0.52)		<0.001 *
						510.3 ± 154.55		
						48.69 ± 36.14 mg		0.001 *
	Control (Port Site Infiltration)		40	1 h		0.00(0.00)	2.25	
				2 h		0.30(0.56)	1.95	0.004 *
				3 h		2.05(0.96)	0.2	<0.001 *
				6 h		3.10(1.19)	−0.85	<0.001 *
				12 h		2.48(0.82)	−0.23	<0.001 *
				24 h		2.25(0.59)		<0.001 *
						292.7 ± 67.03		
						141.8 ± 60.01 mg		0.001 *
23	Altiparmak [77]	2019						
	US-OSTAP Block.							
					96.3 ± 30.8<br>8<br>24<br>24			0.821
				First 24 h		48		
				First 24 h		199.4 ± 27.7		<0.001 *
	US-ESP Block							
					95.5 ± 23.3			0.821
				1 h		4		
				First 12 h		8		
				2nd 12 h		16		
				First 24 h		24		
				First 24 h		139.1 ± 21.9		<0.001 *
24	Baytar [78]	2019						
	US-TAP Block (subcostal)		53					
					1.63 ± 0.23			0.056 *
						63.73 (103.42)		0.187
						83.43(71.25)		0.754

						Visual Analogue Scale		
				0 h		1.33(1.50)	−1.22	0.418
				1 h		0.75(1.29)	−0.64	0.227
				6 h		0.47(0.95)	−0.36	0.734
				12 h		0.20(0.53)	−0.09	0.554
				24 h		0.11(0.37)		0.951
						Dynamic Visual Analogue Scale		
				0 h		2.42(2.10)	−2.1	0.879
				1 h		1.94(1.87)	−1.62	0.720
				6 h		1.38(1.52)	−1.06	0.535
				12 h		1.00(1.28)	−0.68	0.815
				24 h		0.32(0.85)		0.402
	Quadratus Lumborum-Block		54					
					1.67 ± 0.27			0.056 *
						70.00 ± 92.76		0.187
						86.66 ± 67.82		0.754
						Visual Analogue Scale		
				0 h		1.03(1.18)	−0.94	0.418
				1 h		0.70(0.76)	−0.61	0.227
				6 h		0.42(0.74)	−0.33	0.734
				12 h		0.22(0.46)	−0.13	0.554
				24 h		0.09(0.029)		0.951
						Dynamic Visual Analogue Scale		
				0 h		2.42(2.10)	−1.96	0.879
				1 h		1.90(1.49)	−1.44	0.720
				6 h		1.50(1.38)	−1.04	0.535
				12 h		0.92(1.24)	−0.46	0.815
				24 h		0.46(0.88)		0.402
25	Houben [79]	2019						
	US-TAP Block (Levobupivacaine)		26					
						5.6(4.9)		0.71
						4.2(5.8)		0.72
						21.2(14.5)		0.48
	US-TAP Block (Saline)		26					
						6.2(6.4)		0.71
						6.5(10.5)		0.72
						25.2(25.3)		0.48
26	Janjua [80]	2019						
	US-TAP Block, Unilateral		50					
				0 h		2.65(1.60)	0.02	<0.001 *
				1 h		3.20(1.26)	−0.53	<0.001 *
				2 h		3.47(1.62)	−0.8	<0.001 *
				8 h		1.36(0.74)	1.31	<0.001 *
				24 h		2.67(1.47)		<0.001 *
	Control (Port Site Infiltration)		50					
				0 h		4.42(1.11)	−0.5	<0.001 *
				1 h		4.64(1.63)	−0.72	<0.001 *
				2 h		5.08(1.45)	−1.16	<0.001 *
				8 h		1.52(0.59)	2.4	<0.001 *
				24 h		3.92(1.88)		<0.001 *
27	Karnik [81]	2019						
	US-TAP Block		40		Pain scores were presented as Median(Interquartile range values)			
				10 min		2(0–2.5)		0.011 *
				30 min		1.5(0–3)		<0.001 *
				1 h		1.5(0–2)		<0.001 *
				2 h		2(0–2)		0.010 *
				3 h		2(0−2)		0.352
				4 h		0(0–2)		0.267
				6 h		0(0–2)		0.509
					5			0.001
						8		0.001
	Control (Port Sites Infiltration)		40		Pain scores were presented as Median(Interquartile range values)			
				10 min		2(3–4)		0.011 *
				30 min		3(2–5)		<0.001 *
				1 h		2(2–3)		<0.001 *
				2 h		2(1.5–2.5)		0.010 *
				3 h		2(0–2)		0.352

				4 h		2(0–2)		0.267
				6 h		0(0–2)		0.509
					38			0.001 *
						30		0.001 *
28	Khandelwal [82]	2019						
	US-STA Block		40					
				0 h		2.32(0.82)	1.05	0.00 *
				1 h		3.32(1.09)	0.05	0.00 *
				2 h		3.37(1.29)	0.0	0.03 *
				4 h		3.65(1.14)	−0.28	0.00 *
				6 h		3.2(1.09)	0.17	0.03 *
				12 h		2.45(1.13)	0.92	0.32
				24 h		3.37(1.29)		0.76
	Control (Intra-peritoneal Infiltration)		40					
				0 h		3.7(1.5)	−0.9	0.00 *
				1 h		4.2(1.39)	−1.4	0.00 *
				2 h		4.02(1.38)	−1.22	0.03 *
				4 h		4.45(1.17)	−1.65	0.00 *
				6 h		3.77(1.25)	−0.97	0.03 *
				12 h		2.70(1.13)	0.1	0.32
				24 h		2.8(1.50)		0.76
29	Ribeiro [83]	2019						
	US-OSTAP Block (Ropivacaine)		21	0 h		0.71(1.06)	−0.33	0.001 *
				30 min		1.3(1.62)	−0.92	0.035 *
				2 h		1.45(1.65)	−1.07	0.025 *
				4 h		0.90(1.44)	−0.52	0.005 *
				6 h		0.20(0.56)	0.18	0.001 *
				24 h		0.38(0.60)		0.035 *
					122(19.21)			0.575
				First 8 h		4.64(3.38)	1.25	0.001 *
				8–16 h		1.25(2.75)	4.64	0.59
				16–24 h		NA		
				24 h		5.89(6.13)		0.05 *
	US-OSTAP Block (Normal saline)		21	0 h		2.21(1.61)	−0.69	0.001 *
				30 min		2.30(1.35)	−0.78	0.035 *
				2 h		2.52(1.20)	−1.0	0.025 *
				4 h		2.29(1.55)	−0.77	0.005 *
				6 h		1.71(1.05)	−0.19	0.001 *
				24 h		1.52(0.98)		0.035 *
					126.19(21.61)			
				First 8 h		9.52(3.76)	1.78	0.001 *
				8–16 h		0.83(2.14)	10.47	0.59
				16–24 h		0.95(2.11)	10.35	0.042 *
				24 h		11.3(8.03)		0.05 *
30	Siriwardana [84]	2019						
	LAP-TAP Block + port Site infiltration		45					
				6 h		6(2–10)		0.043 *
				12 h		4(2–9)		0.587
				18 h		3(0–7)		0.135
				24 h		2(0–8)		0.188
				36 h		1(0–6)		0.093
						Opioids requirement (Number of doses)		
				6 h		1(0–1)		0.021 *
				12 h		0(0–6)		0.414
				18 h		0(0–3)		0.715
				24 h		0(0–4)		0.267
				36 h		0(0–4)		0.671
	Control (Port Site Infiltration)		45					
				6 h		5(0–9)		0.043 *
				12 h		4(1–7)		0.587
				18 h		2(0–6)		0.135
				24 h		2(0–6)		0.188

				36 h	0(0–5)		0.093
					Opioids requirement (Number of doses)		
				6 h	1(0–1)		0.021 *
				12 h	0(0–6)		0.414
				18 h	0(0–2)		0.715
				24 h	0(0–2)		0.267
				36 h	0(0–2)		0.671
31	Wu [85]	2019					
	LAI Group		60	The three groups did not differ in pain scores at each time point within the 48 h period.			
	TL-Group		60	The three groups did not differ in pain scores at each time point within the 48 h period.			
	TR-Group		60	The three groups did not differ in pain scores at each time point within the 48 h period.			
32	Arik [86]	2020					
	TAP Block, sub-costal		24		Post-operative Pain scores at Rest		
				1 h	3.29(2.1)	−2.46	0.001 *
				3 h	1.87(1.5)	−1.04	0.001 *
				6 h	1.70(1.8)	−0.87	0.001 *
				12 h	1.20(1.2)	−0.37	0.001 *
				24 h	0.83(1.0)		0.001 *
					Post-operative Pain score during Cough		
				1 h	3.83(2.2)	−2.42	0.001 *
				3 h	2.45(1.7)	−1.04	0.001 *
				6 h	2.38(1.9)	−0.97	0.001 *
				12 h	2.04(1.5)	−0.63	0.001 *
				24 h	1.41(1.3)		0.001 *
	Local Anaesthetic Infiltration (Port Sites)		24		Post-operative Pain scores at rest		
				1 h	4.95(2.1)	−3.66	0.001 *
				3 h	2.54(1.7)	−1.25	0.001 *
				6 h	2.29(1.9)	−1.0	0.001 *
				12 h	2.20(1.7)	−0.91	0.001 *
				24 h	1.29(1.2)		0.001 *
					1(4.2)		
					Post-operative Pain score during Cough		
				1 h	5.87(2.2)	−4.0	0.001 *
				3 h	3.50(2.0)	−1.63	0.001 *
				6 h	3.16(2.2)	−1.29	0.001 *
				12 h	2.95(1.8)	−1.08	0.001 *
				24 h	1.87(1.0)		0.001 *
	Control (IV-PCA + GA)		24		Post-operative Pain scores at rest		
				1 h	5.7(2.4)	−4.2	0.001 *
				3 h	2.87(2.2)	−1.37	0.001 *
				6 h	2.62(1.6)	−1.12	0.001 *
				12 h	2.70(2.0)	−1.2	0.001 *
				24 h	1.50(1.50)		0.001 *
					2(8.3)		
					Post-operative Pain score during Cough		
				1 h	6.33(2.3)	−4.46	0.001 *
				3 h	3.20(2.0)	−1.33	0.001 *
				6 h	3.33(1.6)	−1.46	0.001 *
				12 h	3.45(2.4)	−1.58	0.001 *
				24 h	1.87(1.5)		0.001 *
					3(2.5)		
33	Kharbuja [87]	2020					
	US-TAP Block						
				0 h	3.10(0.48)	−0.43	0.044 *
				1 h	2.87(0.81)	−0.2	0.001 *
				2 h	3.23(1.06)	−0.56	0.001 *
				4 h	3.33(1.18)	−0.66	0.001 *
				8 h	3.37(0.90)	−0.7	0.001 *
				16 h	3.10(0.75)	−0.43	0.001 *
				24 h	2.67(0.95)		0.001 *
					3.36(2.09)		0.001 *
					136(66.31)		0.001 *
	Control (Port Site Infiltration)						
				0 h	3.37(0.49)	0.46	0.044 *
				1 h	3.87(1.07)	−0.04	0.001 *
				2 h	4.30(0.988)	−0.47	0.001 *
				4 h	4.37(1.12)	−0.54	0.001 *
				8 h	4.87(1.00)	−1.04	0.001 *
				16 h	4.27(0.94)	−0.44	0.001 *

				24 h	3.83(0.83)		0.001 *
					1.73(1.60)		0.001 *
					202(80.58)		0.001 *
34	Liang [88]	2020					
	Group H (0.75% Ropivacaine)		30				
					PACU *n* (%): 11(36.7%)<br>Ward *n* (%): 4(13.3%)		
	Group M (0.5% Ropivacaine)		30				
					PACU *n* (%): 12(40%)<br>Ward *n* (%): 3(10%)		
	Group L (0.25% Ropivacaine)		30				
					PACU *n* (%): 10(33.3%)<br>Ward *n* (%): 5(16.7%)		
	Group Control (0.9% normal saline)		30				
					PACU *n* (%): 21(70%)<br>Ward *n* (%): 13(43.3%)		
35	Abdelfatah [89]	2021					
	US-TAP-Block group 1 (Bupivacaine + Esmolol)						
				Arrival to PACU	0.87(1.01)	2.16	0.010 *
				1 h	0.97(0.85)	2.06	0.034 *
				2 h	1.67(0.92)	1.36	0.083
				6 h	2.63(1.38)	0.4	0.286
				12 h	3.07(1.53)	−0.04	0.662
				24 h	3.03(1.47)		0.437
					12.91(6.16)		0.148
					5.83(2.87)		0.204
	US-TAP-Block group 2 (Bupivacaine +Isotonic saline)						
				Arrival to PACU	0.83(1.26)	2.5	0.010 *
				1 h	1.7(1.64)	1.63	0.034 *
				2 h	2.03(0.67)	1.3	0.083
				6 h	3.0(1.26)	0.33	0.286
				12 h	3.23(1.41)	0.1	0.662
				24 h	3.33(1.49)		0.437
					9.13(6.69)		0.148
					7.5(3.65)		0.204
36	Ergin [90]	2021					
	TAI-Group		40				
				1 h	3.08(2.75)	−1.33	0.001 *
				2 h	2.6 (2.05)	−0.85	0.001 *
				4 h	2.43(2.16)	−0.68	0.001 *
				6 h	2.2 (2.17)	−0.45	0.001 *
				12 h	2.25(2.25)	−0.5	0.001 *
				24 h	1.75(1.9)		0.001 *
	TAPB-Group		40				
				1 h	3.3(2.44)	−2.17	0.001 *
				2 h	2.43(1.62)	−1.3	0.001 *
				4 h	2.3(1.9)	−1.17	0.001 *
				6 h	2.2(2.19)	−1.07	0.001 *
				12 h	1.23(1.27)	−0.1	0.001 *
				24 h	1.13(1.07)		0.001 *
	IPLA-Group		40				
				1 h	4.7(2.5)	−1.77	0.001 *
				2 h	4.15(1.83)	−1.22	0.001 *
				4 h	4.0(2.18)	−1.07	0.001 *
				6 h	3.53(2.14)	−0.6	0.001 *
				12 h	3.23(1.95)	−0.3	0.001 *
				24 h	2.93(1.86)		0.001 *
	Control (Sham)		40				
				1 h	7.03(2.18)	−2.88	0.001 *
				2 h	6.45(1.71)	−1.3	0.001 *

				4 h			5.35(1.66)	−1.2	0.001 *
				6 h			5.0(1.68)	−0.85	0.001 *
				12 h			4.73(2.1)	−0.58	0.001 *
				24 h			4.15(2.12)		0.001 *
37	Jung [91]	2021							
	BD-TAP Block								
				2 h			3(2–4.75)		0.024 *
							4(3–6)		0.047 *
							4.2(2.1)		0.013 *
				6 h			3(2–4)		0.442
							3.9(1.9)	−1.0	0.281
							3(3–4)		0.218
				12 h			3(2–4)		0.721
							3(3–4)		0.753
							3(2–4.75)		0.771
				24 h			2(2–3)		0.981
							3(2–4)		0.719
							3(2–3)		0.531
				48 h			2(1–3)		0.863
							2.9(1.5)		646
							2(2–3)		0.653
					593.5(507–700)				0.921
					181.5(105–286)				0.771
					2.5(2–3)				0.184
	Control (Sham)								
				2 h			4.5(3–6)		0.024 *
							6(4–8)		0.047
							5.7(2.5)		0.013 *
				6 h			3.0(2–4.8)		0.442
							4.4(2.2)	−1.3	0.281
							4(2–6)		0.218
				12 h			3(2–4)		0.721
							4(2–5)		0.753
							3(2–5)		0.771
				24 h			2(1.3–3.8)		0.981
							3(2–5)		0.719
							3(2–4)		0.531
				48 h			2(1–3)		0.863
							3.1(1.7)		646
							3(1–3)		0.653
					605.5(500–746)				0.921
					189(130–283)				0.771
					3.0(2–4)				0.184
38	Sahu [92]	2021							
	US-ESP-Block (at T7)		30				Mean values		
				2 h			1.60	−1.5	0.029 *
				4 h			1.17	−1.07	0.001 *
				6 h			0.90	−0.8	0.001 *
				8 h			0.33	−0.23	0.001 *
				10 h			0.60	−0.5	0.001 *
				12 h			0.83	−0.73	0.001 *
				14 h			0.83	−0.73	0.001 *
				16 h			0.20	−0.1	0.001 *
				18 h			0.13	−0.03	0.001 *
				20 h			0.17	−0.07	0.001 *
				22 h			0.10	0	0.001 *
				24 h			0.10		0.001 *
						6.9(1.8)			0.174
	OSTAP-Block (Bilateral)		30						
				2 h			2.27	−01.57	0.029 *
				4 h			2.73	−2.03	0.001 *
				6 h			2.33	−1.63	0.001 *
				8 h			1.80	−1.1	0.001 *
				10 h			1.87	−1.17	0.001 *
				12 h			2.23	−1.53	0.001 *
				14 h			1.70	−1.0	0.001 *
				16 h			1.67	−0.97	0.001 *

				18 h		1.23	−0.53	0.001 *
				20 h		1.07	−0.37	0.001 *
				22 h		1.10	−0.4	0.001 *
				24 h		0.70		0.001 *
					7.6(2.3)			0.174
39	Saravanan [93]	2021						
	US-Modified BRILMA-Block		30					
						5.67(1.98)		0.317
						759.33(80.29)		0.295
	US-TAP-Block		30					
						5.17(1.85)		0.317
						854(93.01)		0.295
40	Vindal [94]	2021			VAS (REST) Median IQR			
	LAP-TAP-Block		50					
				3 h		4(2)		0.577
				6 h		3(1)		0.187
				24 h		2.5(1)		0.146
				Dis-charge		1(1)		0.05 *
				1-week post-op		1(1)		0.001 *
					VAS (Coughing) Median IQR			
				3 h		5(1)		0.001 *
				6 h		4(1)		0.001 *
				24 h		3(1)		0.001 *
				Dis-charge		1.5(1)		0.001 *
				1-week post-op		1(1)		0.001 *
						14(28%)		0.05 *
	Control (Port Site Infiltration)		50		VAS (REST) Median IQR			
				3 h		4(2)		0.577
				6 h		3(1)		0.187
				24 h		3(1)		0.146
				Dis-charge		2(1)		0.05
				1-week post-op		1(1)		0.001 *
					VAS (Coughing) Median IQR			
				3 h		5(1)		0.001 *
				6 h		5(1)		0.001 *
				24 h		3(1)		0.001 *
				Dis-charge		3(1)		0.001 *
				1-week post-op		1(0)		0.001 *
						26(52%)		0.05 *
41	Priyanka [95]	2022						
	US-TAP Block (Pre-induction)							
				30 min		3.18(1.04)	0.29	7.36×10^{-7}
				1 h		0.03(0.84)	4.44	1.73×10^{-6}
				2 h		3.13(1.00)	0.34	2.82×10^{-5}
				4 h		3.38(1.00)	0.09	3.69×10^{-6}
				8 h		3.42(1.00)	0.05	4.93×10^{-6}
				12 h		3.53(1.01)	−0.06	2.83×10^{-4}
				24 h		3.47(0.81)		5.92×10^{-5}
	US-TAP Block (Post-surgery)							
				30 min		4.96(1.08)	−0.33	7.36×10^{-7}
				1 h		4.30(1.19)	0.33	1.73×10^{-6}
				2 h		4.54(1.30)	0.09	2.82×10^{-5}
				4 h		4.69(1.28)	−0.06	3.69×10^{-6}
				8 h		4.39(1.16)	0.24	4.93×10^{-6}

				12 h		4.71(1.29)	−0.08	2.83×10^{-4}
				24 h		4.63(1.18)		5.92×10^{-5}
42	Emile [96]	2022						
	US-TAP Block							
				2 h		4.4(1.3)	−3.0	0.0001 *
				6 h		2.9(0.8)	−1.5	0.0001 *
				12 h		1.9(0.7)	−0.5	0.0001 *
				24 h		1.4(0.7)		0.0001 *
				24 h		1027.8(166.7)		0.0001 *
						5.1(1.2)		0.0001 *
						1(1–2)		0.0001 *
	LSTAP Block							
				2 h		4.8(1.2)	−3.5	0.0001 *
				6 h		3.3(1.1)	−2.0	0.0001 *
				12 h		2.1(0.7)	−0.8	0.0001 *
				24 h		1.3(0.5)		0.0001 *
						1055.5(232.3)		0.0001 *
						5.4(1.0)		0.0001 *
						1(1–2)		0.0001 *
	Control (GA only)							
				2 h		6.4(1.7)	−4.2	0.0001 *
				6 h		6.7(1.5)	−4.5	0.0001 *
				12 h		3.2(1.1)	−1.0	0.0001 *
				24 h		2.2(0.9)		0.0001 *
					1657.9(668.8)			.0001 *
					4.1(1.2)			.0001 *
					2(1–2)			.0001 *
43	Fargaly [97]	2022						
	US-TAP Block Group		25		Median (IQR)			
				30 min		2(2–3)		0.084
				2 h		3(3–3)		0.004 *
				4 h		33–4)		0.008 *
				6 h		4(4–5)		0.0001 *
				12 h		5(4–6)		0.0001 *
				24 h		4(4–5)		0.026 *
						Yes = 18, 72%<br>No = 7, 28%		0.239
					Median (1QR) =>	8(6–24)		0.001 *
						6(6–9)		0.0001 *
	Quadratus Lumborum (QLB-Group)		25		Median (IQR)			
				30 min		2(2–2)		0.084
				2 h		2(2–3)		0.004 *
				4 h		3(3–3)		0.008 *
				6 h		33–4)		0.0001 *
				12 h		43–4)		0.0001 *
				24 h		4(3–4)		0.026 *
						Yes = 14, 56%<br>No = 11, 44%		0.239
					Median (1QR) =>	17(12–24)		0.001 *
						3(3–6)		0.0001 *
44	Han [98]	2022						
	US-TAPB Block (Ropivacaine + Tropisetron + Normal saline)		60		The US-TAPB block group had significantly lower resting VAS values than the S and N groups at 2, 6, 12, 24, and 48 h postoperatively ($p < 0.05$). Postoperatively, dynamic VAS was significantly lower in the US-TAPB block group ($p < 0.05$) than in the S and N groups.			
	Group S (Sufentanil + Tropisetron + Normal saline)		60		The US-TAPB block group had significantly lower resting VAS values than the S and N groups at 2, 6, 12, 24, and 48 h postoperatively ($p < 0.05$). Postoperatively, dynamic VAS was significantly lower in the US-TAPB block group ($p < 0.05$) than in the S and N groups.			
	Group N (Nalbuphine + Tropisetron + Normal saline)		60		The US-TAPB block group had significantly lower resting VAS values than the S and N groups at 2, 6, 12, 24, and 48 h postoperatively ($p < 0.05$). Postoperatively, dynamic VAS was significantly lower in the US-TAPB block group ($p < 0.05$) than in the S and N groups.			
45	Lee [99]	2022						
	US-TAP Block (0.375% Ropivacaine)		27		Number (%)			
				1 h		15(55.6%)		0.652
				8 h		0(0.0%)		0.002 *
				24 h		4(14.8%)		0.728

	US-TAP Block (normal saline)		26		Number (%)		
				1 h	17(65.4%)		0.652
				8 h	8(30.8%)		0.002 *
				24 h	5(19.2%)		0.728
46	Ozciftci [100]	2022					
	US-TAP Block, unilateral (right side)		30		VNRS at Rest		
				1 h	3.10(1.61)	−1.87	0.001 *
				2 h	2.70(1.21)	−1.47	0.001 *
				4 h	1.83(0.83)	−0.6	0.001 *
				8 h	1.83(0.83)	−0.6	0.002 *
				12 h	1.40(0.77)	−0.17	0.009 *
				24 h	1.23(0.68)		0.001 *
					VNRS (Cough)		
				1 h	3.37(1.77)	−1.57	0.001 *
				2 h	5.23(2.06)	−3.43	0.001 *
				4 h	2.73(1.34)	−0.93	0.014 *
				8 h	2.57(1.28)	−0.77	0.001 *
				12 h	2.0(1.20)	−0.2	0.001 *
				24 h	1.8(1.01)		0.001 *
	US-TAP Block, bilateral		30		VNRS at Rest		
				1 h	3.33(1.06)	−2.1	0.001 *
				2 h	2.87(1.22)	−1.64	0.001 *
				4 h	2.17(0.79)	−0.94	0.001 *
				8 h	1.73(0.74)	−0.5	0.002 *
				12 h	1.63(0.89)	−0.4	0.009 *
				24 h	1.23(0.90)		0.001 *
					VNRS (Cough)		
				1 h	3.47(1.48)	−1.9	0.001 *
				2 h	3.20(1.35)	−1.63	0.001 *
				4 h	2.53(1.01)	−0.96	0.014 *
				8 h	2.0(0.74)	−0.43	0.001 *
				12 h	1.83(1.02)	−0.26	0.001 *
				24 h	1.57(1.14)		0.001 *
	Control (GA only)		30		VNRS at Rest		
				1 h	6.03(1.52)	−3.93	0.001 *
				2 h	4.50(1.71)	−2.4	0.001 *
				4 h	2.80(1.19)	−0.7	0.001 *
				8 h	2.60(1.13)	−0.5	0.002 *
				12 h	2.17(1.02)	−0.07	0.009 *
				24 h	2.10(1.09)		0.001 *
					VNRS (Cough)		
				1 h	6.33(1.81)	−3.26	0.001 *
				2 h	3.33(1.69)	−0.26	0.001 *
				4 h	3.63(1.59)	−0.56	0.014 *
				8 h	3.57(1.46)	−0.5	0.001 *
				12 h	3.13(1.51)	−0.06	0.001 *
				24 h	3.07(1.70)		0.001 *
47	Paudel [101]						
	US-TAP Block		30	US-TAP Block and local infiltration had significantly different postoperative pain scores (VAS) at 0, 2, 6, 12, and 24 h. The VAS score is significantly higher in the local infiltration group than in the US-TAP block group ($p < 0.001$).			
				24 h	24.67(24.17)		0.001 *
					3.45(1.12)		0.001 *
	Control (port site infiltration)		30	US-TAP Block and local infiltration had significantly different postoperative pain scores (VAS) at 0, 2, 6, 12, and 24 h. The VAS score is significantly higher in the local infiltration group than in the US-TAP block group ($p < 0.001$).			
				24 h	3.45(1.12)		0.001 *
					1.75(0.70)		0.001 *
48	Rahimzadeh [102]	2022					
	US-TAP Block (post-surgery)	38					
					34.21(3.64)		0.518
					2.22(1.20)		0.089
	US-TAP Block (after induction)	38					
					32.11(2.67)		0.581
					5.80(2.68)		0.089

Time = Time after the surgical operation; GA = General Anaesthesia; IV = Intravenously; IQR = Interquartile Range; LAI-Group = received pre-operational administration of 0.5% Ropivacaine plus 1mcg of Dexamethasone at the trocar entrance; TL-Group = received pre-operational administration of 0.5% Ropivacaine plus

1mcg of Dexamethasone at the trocar entrance alongside posterior US-TAP block; TR-Group = US-TAP block combined with rectus sheath block; BD-TAP = bilateral dual transversus abdominis plane; VNRS = Verbal Numerical Rating Scale; PCA = Patient Controlled Analgesia; IV-PCA = Intravenous Patient Controlled Analgesia PCIA = Patient Controlled Intravenous Analgesia; NAS = Numeric Analogue Scale; VAS = Visual Analog Scale; NR = Not Reported; NRS = Numerical Rating Scale; US-OSTAP = Ultrasound-guided oblique subcostal tranversus abdominis plane; TAP block = tranversus abdominis plane block; OSTAP = oblique subcostal tranversus abdominis; US-STA = Subcostal Ultrasound Tranversus Abdominis; Subcostal Tranversus Abdominis; US-Posterior TAP = Ultrasound guided posterior tranversus abdominis plane block; Subcostal TAP block: subcostal tranversus abdominis plane block; US-ESP = Ultrasound-guided erector spinae plane; US-STA Block = Ultrasound Guided Subcostal Tranversus Abdominis; LAP-TAP = Laparoscopic tranversus abdominis plane; US-TAPB = Ultrasound-guided tranversus abdominis plane block; RSB = Rectus Sheath Block; LAI = Local Anaesthetic Infiltration; TAPB = tranversus abdominis plane block; IPLA = Intraperitoneal Local Anaesthetic Injection; Modified BRILMA Block = Blocking the Branches of Intercostal nerves at the Level of Mid-Axillary line; LSTAP = Laparoscopic Subcostal TA; * p-value less than 0.05.
